# Psychosis in Alzheimer's Disease Is Associated With Increased Excitatory Neuron Vulnerability and Post-transcriptional Mechanisms Altering Synaptic Protein Levels

**DOI:** 10.3389/fneur.2022.778419

**Published:** 2022-03-02

**Authors:** Michael R. DeChellis-Marks, Yue Wei, Ying Ding, Cody M. Wolfe, Joshua M. Krivinko, Matthew L. MacDonald, Oscar L. Lopez, Robert A. Sweet, Julia Kofler

**Affiliations:** ^1^Department of Psychiatry, University of Pittsburgh School of Medicine, Pittsburgh, PA, United States; ^2^Department of Biostatistics, University of Pittsburgh School of Public Health, Pittsburgh, PA, United States; ^3^Department of Environmental and Occupational Health, University of Pittsburgh School of Public Health, Pittsburgh, PA, United States; ^4^Department of Neurology, University of Pittsburgh School of Medicine, Pittsburgh, PA, United States; ^5^Department of Pathology, University of Pittsburgh School of Medicine, Pittsburgh, PA, United States

**Keywords:** Alzheimer's disease, psychosis, transcriptomic (RNA-seq), post-transcription, postmortem, dorsolateral prefrontal cortex (DLPFC)

## Abstract

Alzheimer's disease with psychosis (AD+P) is a heritable phenotypic variant of the disease which is associated with more rapid cognitive deterioration compared to Alzheimer's disease without psychosis (AD–P). Cognitive decline in AD correlates with synapse loss, and our previous studies suggest that those with AD+P have a differentially affected synaptic proteome relative to those with AD–P. In this study, we utilized RNA-sequencing of dorsolateral prefrontal cortex (DLPFC) in a cohort of 80 AD cases to evaluate novel transcriptomic signatures that may confer risk of psychosis in AD. We found that AD+P was associated with a 9% reduction in excitatory neuron proportion compared to AD–P [Mean (SD) AD+P 0.295 (0.061); AD–P 0.324 (0.052), *p* = 0.026]. mRNA levels contributed only modestly to altered synaptic proteins in AD+P relative to AD–P. Instead, network analysis identified altered expression of gene modules from protein ubiquitination, unfolded protein response, eukaryotic initiation factor 2 (EIF2) signaling and endoplasmic reticulum stress pathways in AD+P. We previously found that neuropathologies account for ~18% of the variance in the occurrence of psychosis in AD. Further inclusion of cell type proportions and differentially expressed modules increased the percent of the variance in psychosis occurrence accounted for in our AD cohort to 67.5%.

## Introduction

Psychosis in Alzheimer's disease (AD with psychosis, AD+P), defined as the presence of delusions and/or hallucinations, is a heritable phenotype, comprising 40–60% of those affected by AD (1). AD+P is associated with hastened cognitive decline and elevated mortality compared to AD without psychosis (AD–P) (2). AD+P patients endure greater cognitive impairment, are more likely to be institutionalized during illness, and are affected by neuropsychiatric disturbances (e.g., aggression, agitation, depression) at higher rates compared to AD–P counterparts (3, 4). Because psychosis in AD is heritable, it is likely to have a distinct neurobiology (5–9). Further elucidating the neurobiological basis of risk toward psychosis in AD may therefore provide leads to innovative preventions or treatments.

AD neuropathology is defined by fibrillar deposits of amyloid beta and phosphorylated tau. In most cases, however, one or more comorbid neuropathologies (10), including Lewy bodies (11), Transactive response DNA Binding Protein 43 kDa (TDP-43) inclusions (12), and cerebrovascular lesions are also present (13, 14). Previously we set out to establish a comprehensive model of the association of these pathologies with psychosis risk in AD. This stepwise logistical regression identified pathologies that significantly associated with psychosis, including: phosphotau burden, presence of TDP-43 inclusions, an index of microglial activation, and indices of ischemia. However, these neuropathologies only accounted for 18% of the variance in psychosis risk (15). Further, we reported that after accounting for the contribution of neuropathologies, AD+P was associated with lower levels of canonical postsynaptic density proteins in the DLPFC compared to AD–P (15). Synaptic protein levels are potentially impacted by multiple factors including expression levels of their corresponding mRNAs and rates of translation initiation and of protein degradation. In the context of neurodegenerative disease, synaptic protein levels are also strongly impacted by neuron loss (16).

Reduced gray matter volume and reduced indices of synaptic function in AD+P relative to AD–P have been replicated across multiple cerebral neocortical regions, particularly in bilateral frontal and prefrontal cortices (2). In this study we focused on the dorsolateral prefrontal cortex (DLPFC) of AD+P and AD–P subjects. We utilized RNA-sequencing to identify molecular correlates of the excess neuropathologic burden and altered synaptic proteostasis in AD+P. We found that excitatory neurons, as measured by unique excitatory neuron transcript levels, are more vulnerable in AD+P than in AD–P. In addition, we identified co-expressed mRNA modules that were differentially expressed between AD+P and AD–P when controlling for cell proportion and were enriched for mRNAs regulating protein availability. Our findings suggest that the prior observation of synaptic protein reduction in AD+P relative to AD–P subjects results from contributions of neuronal survival and post-transcriptional regulation.

## Methods

### Subjects

We studied a cohort of 80 Alzheimer's disease subjects (Table 1 and Supplementary Data Sheet: Appendix 1) obtained through the brain bank of the Alzheimer's Disease Research Center (ADRC) at the University of Pittsburgh. All subjects, when living, provided informed consent to participate using protocols approved by the University of Pittsburgh Institutional Review Board. The study of postmortem tissue was approved by the University of Pittsburgh Committee for Oversight of Research and Clinical Training Involving Decedents. Subjects underwent comprehensive evaluations by experienced clinicians in the University of Pittsburgh ADR), including neurologic, neuropsychological, and psychiatric assessments as previously described (17, 18). Psychosis was evaluated with the CERAD behavioral rating scale (CBRS) (19). The CBRS was administered at initial and annual visits and in some subjects between annual visits by telephone (7, 20). Subjects were classified as AD+P if they had any hallucination or delusion symptom (CBRS item # 33–45) for 3 or more days in the previous month at any visit (5, 20–24). Subjects with a preexisting psychotic disorder (e.g., schizophrenia) were excluded from the study. Statistical analyses of demographic and clinical differences between groups used *t*-test or Chi-Squared test as appropriate.

**Table 1:** Demographic, clinical, and tissue characteristics of subjects with Alzheimer's disease with and without psychosis examined by RNA-sequencing.

**Variable**	**Alzheimer's disease without psychosis** **(*****n*** **= 33)**		**Alzheimer's disease with psychosis** **(*****n*** **= 47)**	
	**Mean or total**	**SD or %**	**Mean or total**	**SD or %**
Age (years)	83.7	±7.3	82.0	±6.0
Age at onset (years)^a^	75.6	±8.0	72.1	±6.6
Duration of illness (years)^a^	8.1	±3.0	9.0	±3.3
Postmortem interval (hours)	6.3	±4.0	5.9	±3.9
Tau area ratio	0.1	±0.1	0.1	±0.1
Microvascular lesion sum score	0.3	±0.5	0.2	±0.4
HLA-DR:Iba1 ratio	0.5	±0.7	0.4	±1.3
**Sex**				
Male	21	63.6%	28	59.6%
Female	12	36.4%	19	40.4%
**Braak stage**				
III	5	15.2%	5	10.6%
IV	15	45.5%	18	38.3%
V	13	39.3%	24	51.1%
**APOE-4 status**				
Positive	17	51.5%	29	61.7%
Negative	16	48.5%	18	38.3%
**TDP-43 pathology**				
Positive	18	56.2%	34	72.3%
Negative	15	43.8%	13	27.7%
**Lewy body pathology** ^**b**^				
Positive	12	36.4%	30	63.8%
Negative	21	63.6%	17	36.2%
**Microvascular lesion count**				
0	23	67.7%	36	76.7%
≥1	10	32.3%	11	23.4%
**Antipsychotic use**				
Yes	2	6.1%	9	19.1%
No	31	93.9%	38	80.9%

a *Data unavailable for one subject, Alzheimer's Disease Without Psychosis*.

b *p <0.05*.

### Sample Collection and Neuropathological Assessment

For ADRC subjects, frozen gray matter samples from the right superior frontal gyrus of the DLPFC were retrieved from the ADRC neuropathology core for RNA sequencing. The corresponding formalin-fixed left DLPFC was used for immunostaining and processed for neuropathologic studies as previously described [(15); Supplementary Data Sheet: Appendix 2, 3]. Neuropathologic diagnoses of Alzheimer disease were made according to NIA-Reagan criteria (25), with all cases meeting criteria for intermediate to high probability that their dementia was due to AD.

### Quantitative Immunohistochemistry and Digital Image Analysis

Neuropathological disease burden in the DLPFC was previously assessed in all 80 cases using quantitative immunohistochemistry (15). In short, serial 5 μm thick formalin-fixed, paraffin-embedded tissue sections were immunostained on an automated stainer (Discovery Ultra, Ventana, Tucson, AZ) using the following primary antibodies (Supplementary Data Sheet: Appendix 3): PHF-1, oligomeric tau T22, beta-amyloid NAB228, microglial markers Iba1 (Ionized calcium binding adaptor molecule 1), and HLA-DR (Human Leukocyte Antigen—DR isotype). No counterstaining was performed to ease signal quantification.

Whole slide digital images of the immunostained sections were created using a Mirax MIDI slide scanner (Zeiss, Jena, Germany) at 40× resolution (0.116 micron/pixel). Digital image analysis was performed using NearCyte software (Andrew Lesniak, University of Pittsburgh) as previously described (15). All analyses were done blinded to psychosis status.

### Nucleic Acid Extraction

Frozen tissue was bead-homogenized (Benchmark Scientific, Model No. D1030) in Trizol (Invitrogen; Carlsbad, CA), followed by phase separation using chloroform (20% vol:vol). RNA was then precipitated with isopropanol, washed in 75% ethanol, and resuspended in DEPC-treated water. RNA concentration and purity were assessed using Nanodrop optical density reader.

### RNA Sequencing

RNA sequencing was performed at the Next-Generation Sequencing Core at the University of Pennsylvania, Philadelphia, PA. Library preparation was performed using Illumina truSeq stranded total RNA (ribo-Zero) kits, followed by single-end, 100 bp sequencing on a HiSeq4000 sequencer. Because RNA Integrity Number (RIN) values were low for many samples (Supplementary Data Sheet Table S1), we used RNA-seq read count as our quality screen for sample inclusion. Samples from 85 AD subjects were submitted but 5 cases had extremely low counts of sequence reads (Supplementary Data Sheet Table S1) and were eliminated from further analyses. Neither RIN nor read counts differed significantly between AD+P and AD–P groups in the remaining 80 subjects (*p* = 0.87 and *p* = 0.28, respectively). Sequencing reads were checked using FastQC and aligned to the reference genome (human genome hg38) using TopHat2 (26). RNA-seq reads that had low sequencing quality or mapping quality were filtered out. Gene expressions were then quantified using Cufflinks (27) and a generative statistical model of RNAseq experiments was applied to estimate FPKM (expected Fragments Per Kilobases per Million mapped fragments). A total of 22,440 genes were identified and passed initial QC. After removing genes with missing names or those not quantified in more than 50% samples in both patient groups, 15,346 genes remained (Supplementary Data Sheet Table S2). Missing values of each remaining gene were imputed by the half of the minimum observed value across all samples. We then applied quantile normalization across samples using normalize Quantiles function from R package Limma, and the resulting data were utilized for the downstream analyses (28, 29).

### Cell Type Fraction Estimation

We applied the est_frac function from R package MIND (30) to estimate the cell type proportions in our RNAseq data. It used the non-negative least square deconvolution approach proposed by Wang et al. (31). The signature matrix was taken from the adult single-cell RNAseq data from Darmanis et al. (32), which contains 666 genes and 6 cell types (astrocytes, endothelial cells, microglia, excitatory neurons, inhibitory neurons, and oligodendrocytes). The two-sample *t*-test was then applied to compare each cell type proportion between AD+P and AD–P samples.

### Differential Expression Analysis

Analysis of covariance (ANCOVA) was performed for each gene on the log_2_ transformed scale to compare between AD+P and AD–P, adjusted for neuropathological variables including tau area ratio, TDP-43 pathology status, HLA-DR:Iba1 ratio, microvascular lesion count and sum score. ANCOVA adjustments regarding neuropathological variables were included based on previous data which indicated each was associated with risk of psychosis (15). Analyses also adjusted for the proportion of endothelial cells, oligodendrocytes, and excitatory neurons (which were found to be different or marginally different (*p* < 0.10) between AD+P and AD–P from cell type proportion estimation) (Supplementary Files 1, 2). Other potential confounders (sex, APOE4, antipsychotic drug use) were evaluated for potential effects on gene expression (Supplementary Figure 1, see also Supplementary Data Sheet for Figure Legends). Because none of these three variables demonstrated significant main effects or interactions with psychosis impacting gene expression they were excluded from the ANCOVA models.

The covariate-adjusted fold change, *p*-value and FDR adjusted *q*-value were obtained. We further examined differentially expressed genes within each cell type by adapting CellDMC from R package EpiDISH on our RNAseq data [(33); Supplementary Figure 3, see also Supplementary Data Sheet for Figure Legends]. CellDMC was originally designed for identification of differentially methylated cell types in epigenome-wide association studies through an interaction model. We used the gene expression as the outcome and tested the interaction between cell type proportion and the psychosis status, with the same neuropathological variables adjusted.

### Correlation Analysis

Previously we reported that the mean ratio of synaptic proteins were increased in AD–P relative to AD+P (15). In our RNAseq data, 180 transcripts of the 190 synaptic proteins previously evaluated were present. First, we fit the same ANCOVA model (without cell type proportions adjusted) as in Krivinko et al. (15) for each of these 180 genes and obtained the log_2_ fold change. Then we computed the Spearman's correlation between the previously reported protein-level fold change and the transcriptomic-level fold change for these 180 genes. We further examined whether this protein-transcriptome correlation changed after cell type proportions being adjusted in the RNAseq ANCOVA analysis.

### Gene Co-expression Network Analysis

Gene co-expression network analysis was performed using the R package MEGENA (CRAN) (34). The module size was set to contain at least 15 genes. Pearson correlation was used for constructing the correlation matrix. The number of permutations for calculating FDRs for all correlation pairs and connectivity significance *p*-value was set to 100. After identification of modules and computing their corresponding module eigengenes (MEs), we constructed a model for the presence of psychosis. We first tested all MEs in individual logistic regressions, adjusting for the same neuropathological measures and three cell type proportions as covariates. The final model consisted of all MEs from the individual analyses with *p* < 0.1, and the same neuropathological variables and cell type proportions. The performance of the model is characterized by area under the curve (AUC) and *R*^2^ (i.e., % variance explained) (35). We compared the nested models with and without cell type proportions and/or MEs using the likelihood ratio test, as well as the DeLong's method for comparing AUCs (36), to assess whether including cell type proportions and MEs improve the model fitting and performance.

### Functional Gene Annotation

A gene list was generated by combining all genes that were listed in individual modules which had significant association with AD+P in the multivariate analysis. This list was submitted for *Core Expression Analysis* using Ingenuity Pathway Analysis (IPA, Qiagen). Top canonical pathways with Benjamini-Hochberg *p* < 0.05 and number of genes >4 were identified.

## Results

### Deconvolution of Cell Type Proportions

**Figure 1:**
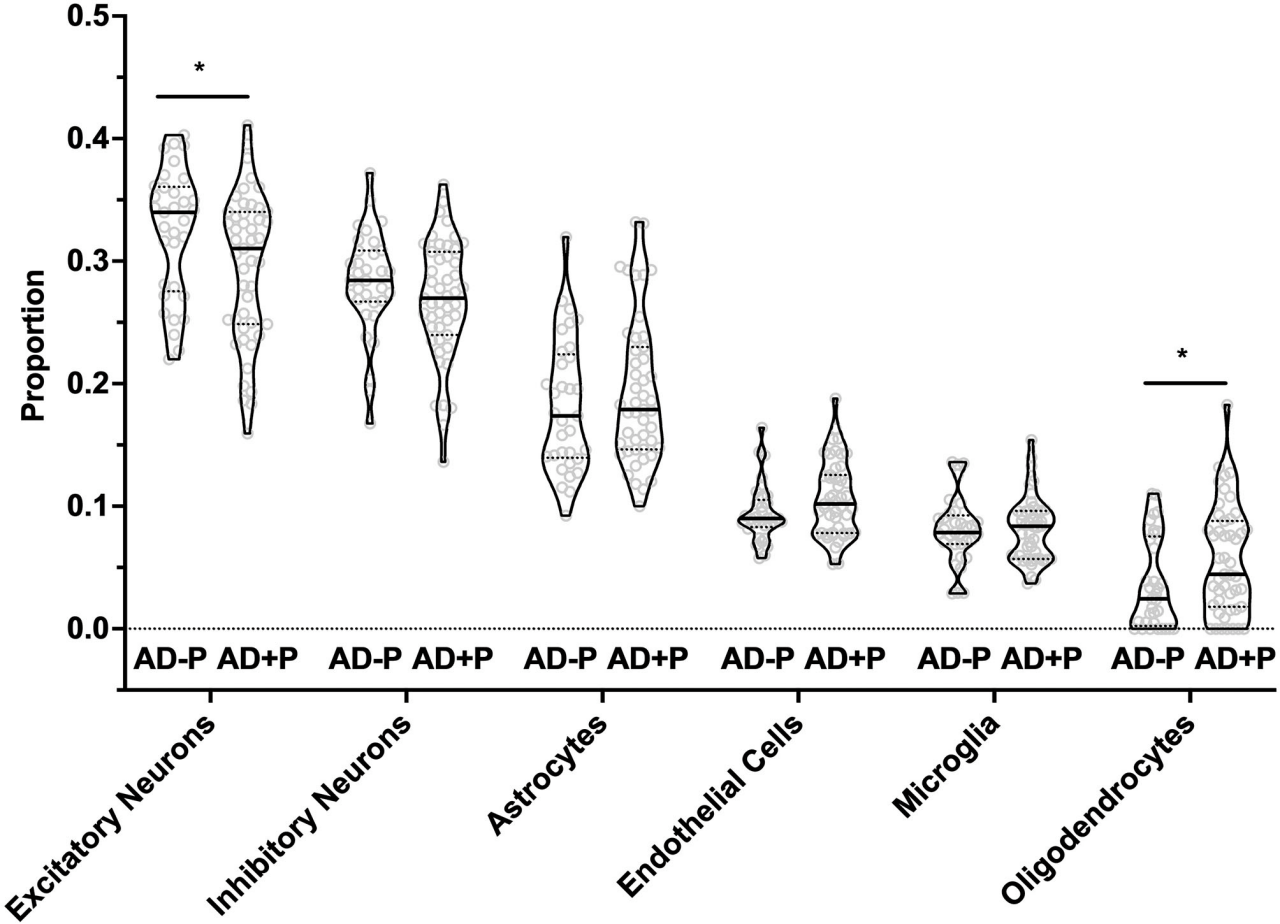
Excitatory Neurons, Oligodendrocytes, and Endothelial Cell proportions are altered in AD+P. Cell type proportion differences between AD+P and AD–P were determined using MIND signature gene matrix (32). Excitatory neuron proportion was significantly reduced in AD+P subjects compared to AD–P (*T* = 2.27 *df* = 75, *p* = 0.026). Conversely, oligodendrocyte (*T* = −2.17, *df* = 76, *p* = 0.033) and endothelial cell (*T* = −1.93, *df* = 78, *p* = 0.057) proportions were increased in AD+P compared to AD–P. Proportions of astrocytes, microglia, and inhibitory neurons did not differ between groups (astrocyte *T* = −0.90, *df* = 73, *p* = 0.372 microglia *T* = −0.03, *df* = 67, *p* = 0.978; inhibitory neuron *T* = 1.40, *df* = 74, *p* = 0.166). *Indicates *p* < 0.05.

Cell type proportion was estimated for astrocytes, endothelial cells, microglia, excitatory neurons, inhibitory neurons, and oligodendrocytes (Figure 1). Notably, there was a decreased proportion of excitatory neurons in AD+P compared to AD–P (*T* = 2.27; *df* = 75, *p* = 0.026). Because the estimates of cell proportion are derive from read counts of multiple RNAs, we examined whether reduced excitatory neuron proportion in AD+P might be an artifact of low RNA-seq read counts, finding no significant association (Supplementary Figure 2, see also Supplementary Data Sheet for Figure Legends). RIN, which may reflect tissue integrity that could also impact cell proportions, was correlated with excitatory neuron proportions (Supplementary Figure 2, see also Supplementary Data Sheet for Figure Legends), but this correlation did not differ between groups (psychosis-by-RIN interaction *p* = 0.41). and thus did not account for the reduced proportions in AD+P. There was a modest inverse correlation of excitatory neuron proportion with tau burden (Supplementary Figure 3, see also Supplementary Data Sheet for Figure Legends). However, the relationship with tau burden was not significant in AD+P, in which group a number of subjects have low excitatory neuron proportions despite modest local tau burden. The relationship of excitatory neuron proportion to other neuropathology measures is shown in Supplementary Figure 3, see also Supplementary Data Sheet for Figure Legends.

The decrease in excitatory neuron proportion was offset by a corresponding increase in the proportion of oligodendrocytes (*T* = −2.17, *df* = 68, *p* = 0.033) and endothelial cells (*T* = −1.93, *df* = 78, *p* = 0.057, see also Supplementary Figure 4, see also Supplementary Data Sheet for Figure Legends). Consistent with our prior report that Iba1 volume fraction was not significantly different between AD+P and AD–P subjects (15), we found no change in microglia proportion (*T* = −0.03, *df* = 67, *p* = 0.978).

### Differential Expression Analysis

Our data set underwent differential expression (DE) analysis with adjustments for neuropathological covariates (15) and the proportions of excitatory neurons, oligodendrocytes, and endothelial cells. We identified 1,077 nominally DE genes (*p* < 0.05), across all cell types (Supplementary Figure 4, see also Supplementary Data Sheet for Figure Legends, Supplementary Data Sheet Table S3A, and Supplementary Files 1, 2). However, none of the 1,077 genes passed an FDR threshold of *q* < 0.1. Additionally, we probed DE genes by cell type. Depending on cell type, we identified a range of 604–1,287 nominally DE genes (Supplementary Data Sheet Table S3B and Supplementary File 3). Two genes passed FDR threshold: *METTL22* (*q* = 0.099) in Inhibitory Neurons and *DKC1* (*q* = 0.072) in Oligodendrocytes.

### Gene Co-expression Network Analysis

Co-expressed genes were clustered into 288 modules (Supplementary File 4). We tested each ME for strength of association with psychosis in a multiple regression model along with neuropathologic covariates and the cell type proportions that differed between AD+P and AD–P (endothelial cells, excitatory neurons, and oligodendrocytes). We identified significant differential expression of 27 MEs between AD–P and AD+P. To avoid redundancy, parent modules that showed significant differential expression were removed, bringing our total to 23 individual modules with *p* < 0.05 (Supplementary File 5). When these were then entered into a multivariate model only 8 modules remained significant (Supplementary Data Sheet Table S4). Functional annotation of the 8 modules identified several significantly enriched canonical pathways (Table 2). These include regulation of protein availability via protein ubiquitination system pathways, unfolded protein response, EIF2 signaling pathways, and endoplasmic reticulum stress pathways.

**Table 2:** Functional annotation analysis of differentially expressed gene modules.

**Canonical pathway**	**Genes identified**	***p***
Protein ubiquitination pathway	ANAPC5, CRYAB, DNAJB6, DNAJC21, HSPAA1, HSPAB1, HSP90B1, HSPA4, HSPA5, HSPA4L, HSPE1, HSPH1, MED20, SUGT1, UBC, UBE2F, UBR2	9.21E-06
Unfolded protein response	ATF4, DDIT3, DNAJB6, DNAJC21, HSP90B1, HSPA4, HSPA5, HSPH1, SCAP	1.58E-04
EIF2 signaling	AGO3, ATF4, DDIT3, EIF3A, EIF4G2, EIF5B, HSPA5, RPL3, RPL8, RPL30, RPL36, RPL37, RPL26L1	2.57E-04
Aldosterone signaling in epithelial cells	CRYAB, DNAJB6, DNAJC21, HSP90AA1, HSP90AB1, HSP90B1, HSPA4, HSPA5, HSPA4L, HSPE1, HSPH1	2.82E-04
Endoplasmic reticulum stress pathway	ATF4, DDIT3, HSP90B1, HSPA5	5.02E-03
Mitotic roles of Polo-like kinase	ANAPC5, HSP90AA1, HSP90AB1, HSP90B1, RAD21	4.74E-02

*Six significantly enriched canonical pathways were determined using IPA core expression analysis*.

### Synaptic Gene Expression

**Figure 2:**
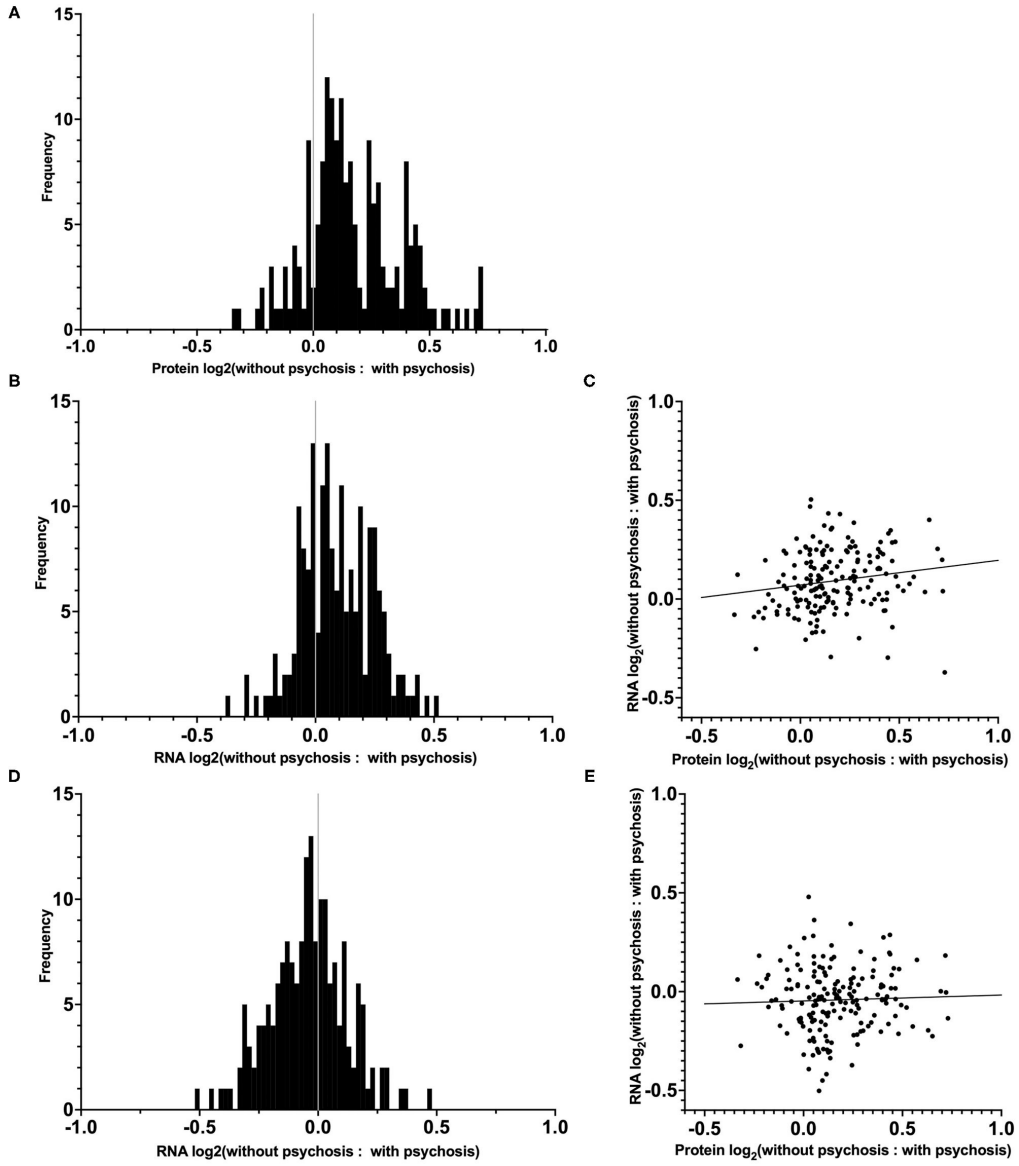
Distribution and correlation of synaptic transcript and protein levels in AD–P relative to AD+P subjects. **(A)** Distributions of log_2_ ratios are shown for 180 synaptic proteins (19) for which corresponding mRNA levels were quantified in the current study. **(B)** Distribution of RNA expression ratios of the 180 synaptic genes identified in our DE analysis, prior to cell type proportion adjustments. The proportion of synaptic transcripts upregulated in AD–P compared to AD+P was 69.4%, compared to 49.6% of non-synaptic transcripts (Chi-square test, *p* = 3.874E-7). **(C)** Correlation between the 180 synaptic protein and transcript expression ratios (AD–P: AD+P, spearman's rho = 0.2257, *p* = 0.0024). **(D)** Analysis of the same synaptic transcripts as in **(B)**, accounting for the contribution of cell type proportions as covariates, eliminates their upregulation in AD–P. **(E)** Inclusion of cell type proportions as covariates in analysis of synaptic transcripts similarly abolishes the correlation between synaptic transcript and protein levels (spearman's rho = 0.0302, *p* = 0.6868).

We had previously reported in a subset of our case cohort that the mean ratio of 190 synaptic proteins was greater in AD–P relative to AD+P (Figure 2A) (15). We evaluated whether decreased synaptic protein abundance in AD+P relative to AD–P subjects results from a relative downregulation of corresponding mRNAs. We successfully measured the abundance of mRNAs corresponding to 180 of the 190 proteins and found that 69.4% of these mRNAs were downregulated in AD+P relative to AD–P. This pattern was not observed in non-synaptic mRNAs (Figure 2B; Chi-square test, *p* = 3.874E-7). However, the correlation between synaptic transcript and protein levels was modest (spearman's rho = 0.2257, *p* = 0.0024, Figure 2C, Supplementary File 6). Given the observed differences in cell type proportions between groups, we asked if alterations in cell type proportions might drive the differences in synaptic gene levels. When cell type proportions were introduced into quantile normalization to estimate transcript expression, there was no longer a relative downregulation of the synaptic genes in AD+P vs. AD–P (Figure 2D) and transcript levels were not correlated with protein levels (spearman's rho = 0.0302, *p* = 0.6868, Figure 2E and Supplementary File 6).

### Models of Psychosis Risk

We previously reported that a combination of neuropathologic variables (immunostaining for phospho-Tau, TDP-43, HLA-DR:Iba1 ratio, and microvascular lesion count plus a summary score of vascular pathologies) together accounted for 18% of the variance in psychosis risk in AD (15). We first undertook to repeat that analysis in the current sample of 80 AD subjects, a subset of the subjects in our prior report. The results of these analyses are shown in Table 3. Neuropathology alone accounted for 11.7% of the variance in psychosis in these subjects (Model 1). The addition of the three cell type proportions improved this to 29.9% with a corresponding improvement in the area under the curve (AUC) (Model 2). Finally, entry of both the 23 significant modules and 3 cell type proportions resulted in an increase in the percent of variance accounted for to 67.5% with an AUC of 0.923 (Model 3). For comparing the three models, the DeLong's AUC test shows a significant *p*-value for Model 2 vs. Model 1 (*p* = 0.021), Model 3 vs. Model 1 (*p* = 0.00014), and for Model 3 vs. Model 2 (*p* = 0.012). On the other hand, the likelihood ratio test shows Model 2 or Model 3 fits the data significantly better than Model 1 (*p* = 0.006 and 0.0008, respectively), and Model 3 fits the data marginally better than Model 2 as well (*p* = 0.063).

**Table 3:** Comparison of models of psychosis.

**Model**	**Neuropathological covariates**	**Modules selected**	**Cell type proportion selected**	***R* ^2^**	**AUC**	**95% CI for AUC**
1	PHF-1 Tau TDP-43 pathology HLA-DR:Iba1 Microvascular lesion Vascular sum score	NA	NA	0.117	0.666	0.540–0.793
2	PHF-1 Tau TDP-43 pathology HLA-DR:Iba1 Microvascular lesion Vascular sum score	NA	Endothelial cells excitatory neurons oligodendrocytes	0.299	0.789	0.680–0.898
3	PHF-1 Tau TDP-43 pathology HLA-DR:Iba1 Microvascular lesion Vascular sum score	23	Endothelial cells excitatory neurons oligodendrocytes	0.675	0.923	0.866–0.980

*Models were constructed using combinations of neuropathological covariates, cell type proportions, and co-expression modules to evaluate their impact on psychosis occurrence. Model 1 used only neuropathological covariates. Model 2 provided a modest improvement by incorporating cell type proportion estimations. Model 3, including neuropathological covariates, cell type proportions, and co-expression modules accounted for the largest percent of variance in occurrence of psychosis*.

## Discussion

We have previously shown that only a minor fraction of the risk for psychosis in AD is attributable to the severity of multiple neuropathological lesions that are present in individuals with AD. We have also previously demonstrated that a decrease in post-synaptic protein abundance was associated with psychosis in AD. Here, we set out to improve our knowledge of neurobiological contributors to psychosis in AD and the mechanisms underlying the synaptic protein decrease by evaluating the transcriptome of AD subjects with and without psychosis. Our data suggests that decreases in excitatory neuron proportion beyond that present in AD subject without psychosis contributes to the underlying neurobiological perturbances associated with psychosis in AD. In addition, network analysis of the transcriptome identified a novel association with gene modules enriched for mRNAs regulating protein availability. Finally, inclusion of cell type proportions and differentially expressed module eigengenes significantly increased the fitness of our models beyond that of neuropathology alone, such that they now accounted for 67.5% of the variance in psychosis presence.

### Excitatory Neurons Are Vulnerable in Alzheimer's Disease With Psychosis

We estimated that there was a decrease of 9% in the excitatory neuron proportion in AD+P subjects compared to AD–P, after controlling for relevant neuropathologic burdens. Because the estimates of cell proportion are derived from measurements of levels of expressed excitatory neuron selective mRNAs, they could reflect either true cell loss or transcript downregulation (although, importantly, they did not appear to result from technical factors, Supplementary Figure 2, see also Supplementary Data Sheet for Figure Legends). Disentangling these two possibilities will require future single-cell RNA sequencing studies of AD+P. However, several lines of evidence support the former interpretation. DLPFC neuron loss occurs in Alzheimer's disease, including within individuals in the mild to moderate stages of disease as in the current study (37). Similarly, we have shown that levels of MAP2 protein, a marker of neuronal survival (38) vary across brain regions in association with the extent of disease-related neuron loss (16). Finally, in a subgroup of the current subjects, and controlling for neuropathologic burden, we reported a 32% decrease in MAP2 protein levels in AD+P subjects compared to AD–P subjects, while both AD phenotypes had significantly less MAP2 peptide levels than unaffected control subjects (15). Thus, it is most likely that both groups of AD subjects in the current study have a decrease in excitatory neuron number in DLPFC, and that the degree of this decrease is greater in AD+P than AD–P. Determining whether the excess loss in AD+P is general among all cortical excitatory neurons or selectively affects a subpopulation that is relatively spared in AD–P, remains an open question.

The above suggests that there is a modest increase in neurodegenerative mechanisms underlying psychosis in AD, due to both an increased burden of select pathologies and due to independent mechanisms. For example, we have recently identified that genetic variation in *SUMF1*, which encodes the formylglycine generating enzyme protein, SUMF1, is associated with psychosis risk in AD in a genome-wide analysis (39). SUMF1 activates multiple lysosomal sulfatases. Genetically driven reductions in SUMF1 expression or function lead to accumulation of glycosaminoglycans and sulfatides (40). Glycosaminoglycans promote formation of insoluble fibrils of amyloid-β and tau, providing a direct link between SUMF1 activity and the primary pathologies of AD (41). However, even in the absence of comorbid AD-related pathologies, SUMF1 knockout is sufficient to generate neuronal cell loss (42).

### Synaptic Transcriptome

Previously, we found that AD+P subjects had a decreased level of synaptic proteins relative to AD–P subjects (15). Our current analysis indicates that mRNA levels contribute only modestly to the synaptic protein impairment in AD+P relative to AD–P. Moreover, because the correlation of transcript levels with protein levels was eliminated when controlling for cell type proportions, the modest contribution of transcript abundance to synaptic protein levels seems to derive from enhanced excitatory neuron loss in AD+P, rather than from a decreased rate of transcription *per se*. Because synaptic proteostasis is a highly dynamic process, where the effects of transcription are modified by local translation, protein trafficking, and degradation, it is likely that these post-transcriptional mechanisms underlie synaptic protein availability in AD+P.

Additionally, our current data suggests a possible role for altered post-transcriptional mechanisms which limit synaptic protein availability in association with psychosis in AD. In our analysis of differentially expressed gene modules, protein ubiquitination pathways, unfolded protein response pathways, eukaryotic initiation factor 2 (EIF2) signaling pathways and endoplasmic reticulum stress pathways were significantly enriched (Table 2). These pathways play a critical role in regulating protein translation and degradation in neurons, including activity-dependent translation and degradation in support of synaptic plasticity (43–49). Thus, our findings suggest that synaptic proteostasis regulation is a key player contributing toward risk of psychosis in AD.

## Conclusion

In this study, we aimed to further nominate neurobiological mechanisms contributing to psychosis in AD. Our current findings suggest that excitatory neurons are selectively vulnerable in the DLPFC of AD+P subjects relative to AD–P. Further, our findings point to the roles of post-transcriptional mechanisms underlying synaptic deficits in AD+P and identify ubiquitination, unfolded protein response, eukaryotic initiation factor 2 (EIF2) signaling, and endoplasmic reticulum stress pathways as possibly contributing to altered synaptic protein abundance in AD+P. Given that the reduction in synaptic indices in AD+P relative to AD–P subjects is common throughout the neocortex (2), the current findings, despite regional variations in gene expression and neuropathology burden, are likely to be conserved in other neocortical regions.

## Data Availability Statement

The datasets presented in this study can be found in online repositories. The names of the repository/repositories and accession number(s) can be found below: https://www.ncbi.nlm.nih.gov/, PRJNA797425.

## Ethics Statement

The studies involving human participants were reviewed and approved by University of Pittsburgh Institutional Review Board. The patients/participants provided their written informed consent to participate in this study.

## Author Contributions

MD-M analyzed the RNA-sequencing data, performed functional annotation analysis, and wrote the manuscript. YW and YD performed raw data processing of RNA-sequencing data, gene network analysis, cell type proportions, and differential gene expression analysis. CW assisted with RNA sample prep and analysis. JK conducted all neuropathologic assessments and with RS designed and oversaw all aspects of the conduct of the study. JK and MM provided the analyses of the synaptic proteome data. RS and OL contributed to the recruitment and assessment of all study subjects while alive. All authors reviewed and approved the final manuscript.

## Funding

This work was supported by NIH grants AG005133 (OL, RS, and JK), AG066468 (OL, RS, and JK) MH116046 (RS, JK, MD-M, MM, YD, and YW), K01 MH107756 (MM).

## Author Disclaimer

The content is solely the responsibility of the authors and does not necessarily represent the official views of the National Institute of Mental Health, the National Institutes of Health, or the United States Government.

## Conflict of Interest

The authors declare that the research was conducted in the absence of any commercial or financial relationships that could be construed as a potential conflict of interest.

## Publisher's Note

All claims expressed in this article are solely those of the authors and do not necessarily represent those of their affiliated organizations, or those of the publisher, the editors and the reviewers. Any product that may be evaluated in this article, or claim that may be made by its manufacturer, is not guaranteed or endorsed by the publisher.
