# Supplementary material for: Psychosis in Alzheimer's Disease Is Associated With Increased Excitatory Neuron Vulnerability and Post-transcriptional Mechanisms Altering Synaptic Protein Levels
Source: Front Neurol. 2022 Mar 2;13:778419. doi: 10.3389/fneur.2022.778419 (PMC8925864; doi:10.3389/fneur.2022.778419)
Supplement: Supplementary file 7 [file Data_Sheet_1.DOCX]

Supplementary Material

# Supplementary Methods

## Appendix 1. Subjects

We studied a total of 80 AD subjects (Table 1) obtained through the brain bank of the Alzheimer Disease Research Center (ADRC) at the University of Pittsburgh, using protocols approved by the University of Pittsburgh Institutional Review Board and Committee for Oversight of Research and Clinical Training Involving Decedents. All cases coming to autopsy between 1993 and 2014 with a primary neuropathologic diagnosis of Alzheimer’s disease and a Braak stage between 3-5 were included in the study. End-stage cases, as defined by a Braak stage of 6, were excluded, as clinically the greatest increase in onset of psychosis in AD occurs between early and middle disease stages [1; 2].

## Appendix 2. Sample Collection and Neuropathological Assessment

For ADRC subjects, postmortem interval (PMI) was recorded at the time of brain removal. At autopsy, the brain was removed intact, examined grossly, and divided in the midsagittal plane. Gray matter samples from the right superior frontal gyrus of the DLPFC were dissected and frozen at −80 °C. The left hemibrain was immersion fixed in 10% buffered formalin for at least one week, sectioned into 1.0 cm coronal slabs, and sampled according to Consortium to Establish a Registry for Alzheimer’s Disease (CERAD) protocol for neuropathological diagnosis of AD [3] or, since 2012, following National Institute of Aging – Alzheimer’s Association (NIA-AA) guidelines [4]. AD pathology was evaluated using the modified Bielschowsky silver stain [5] and immunohistochemical staining for tau and amyloid β. Neuritic plaque density was assessed according to CERAD criteria [3]; distribution of tau pathology was classified according to Braak stages [6]. Neuropathologic diagnoses of Alzheimer disease were made according to NIA-Reagan criteria [7], with all cases meeting criteria for intermediate to high probability that their dementia was due to AD. Lewy body pathology was initially assessed in amygdala, brainstem and olfactory bulb, and if positive, further evaluated in limbic and neocortical sections following consensus guidelines [4; 8]. Immunohistochemical staining for phospho-TDP-43 was performed on sections of amygdala, hippocampus, mesial temporal cortex and middle frontal gyrus as previously described [9]. Sections were evaluated for the absence or presence of TDP-43 positive neuronal cytoplasmic inclusions, neuronal intranuclear inclusions and dystrophic neurites. Since our previous analyses did not reveal any associations of AD+P with disease stages of Lewy body or TDP-43 pathology [10], we continued to stratify these two proteinopathies as positive or negative, whereby all cases with any level of pathology were classified as positive and cases with complete absence of either one of these proteinopathies as negative.

Assessment of vascular pathology included atherosclerosis of the circle of Willis, arteriolosclerosis in frontal white matter and cerebral amyloid angiopathy in DLPFC. Each was rated as none (0), mild (1), moderate (2) or severe (3), and a sum score was generated by adding the three individual scores. Microvascular lesions (MVL) were defined as remote microinfarcts/microhemorrhages not seen on gross examination and less than 1.0 cm in size. MVLs were enumerated in standardized sections [4] of middle frontal gyrus (DLPFC), superior and middle temporal gyrus, inferior parietal lobule, occipital cortex (BA 17/18), basal ganglia at level of anterior commissure, and thalamus at the level of the subthalamic nucleus to create MVL counts.

## Appendix 3. Quantitative Immunohistochemistry and digital image analysis

Neuropathological disease burden in the DLPFC was previously assessed in all 80 cases using quantitative immunohistochemistry [10]. In short, serial 5 μm thick formalin-fixed, paraffin-embedded tissue sections were immunostained on an automated stainer (Discovery Ultra, Ventana, Tucson, AZ) using the following primary antibodies: PHF-1 (1:1000, kindly provided by Peter Davies), oligomeric tau T22 (1:500, EMD Millipore, Billerica, MA), beta-amyloid NAB228 (1:4000, Cell Signaling Technology, Danvers, MA, after 40 min pretreatment with 90% formic acid), and microglial markers Iba1 (Ionized calcium binding adaptor molecule 1) (1:500, Wako, Richmond, VA) and HLA-DR (Human Leukocyte Antigen – DR isotype) (1:100, Dako, Agilent Technologies, Santa Clara, CA). Except for beta-amyloid, slides for all other stains were pretreated with Discovery CC1 solution, a Tris based buffer with a slightly basic pH (Ventana Medical Systems, Tucson, AZ). All slides were developed using a multimeric HRP/DAB detection system (Ventana Medical Systems, Tucson, AZ). No counterstaining was performed to ease signal quantification.

Whole slide digital images of the immunostained sections were created using a Mirax MIDI slide scanner (Zeiss, Jena, Germany) at 40x resolution (0.116 micron/pixel). Digital image analysis was performed using NearCyte software (Andrew Lesniak, University of Pittsburgh). For each section, 4 rectangular regions of interest (ROI) of 4mm^2^ were created. These ROIs were defined to span the entire cortical thickness and were preferentially placed midway along the gyral axis to avoid tangentially cut cortical regions. Minor manual adjustments were made to adapt to curvatures and irregularities in the cortical ribbon. Once placed for the first analyzed stain (PHF-1), the same ROIs were re-used for all subsequent stains. If tissue folds or other artifacts prevented placement in the same location, the ROI was moved to an acceptable site as close as possible to the original location. For quantitative image analysis, thresholds for signal positivity were optimized manually for each stain and then maintained constant throughout the analysis of all slides. Signals from all four ROIs were integrated into two outcome variables: area ratio (= positive area/entire field area) and mean signal intensity. For HLA-DR and Iba1 stains, an additional variable, the HLA-DR/Iba1 ratio was derived to normalize microglial activation (HLA-DR) to microglial density (Iba1). All analyses were done blinded to psychosis status.

# Supplementary References

[1] S.A. Ropacki, and D.V. Jeste, Epidemiology of and Risk Factors for Psychosis of Alzheimer’s Disease: A Review of 55 Studies Published From 1990 to 2003. American Journal of Psychiatry 162 (2005) 2022-2030.

[2] O.L. Lopez, J.T. Becker, R.A. Sweet, W. Klunk, D.I. Kaufer, J. Saxton, et al., Psychiatric Symptoms Vary With the Severity of Dementia in Probable Alzheimer's Disease. The Journal of Neuropsychiatry and Clinical Neurosciences 15 (2003) 346-353.

[3] S.S. Mirra, A. Heyman, D. McKeel, S.M. Sumi, B.J. Crain, L.M. Brownlee, et al., The Consortium to Establish a Registry for Alzheimer&#039;s Disease (CERAD). Neurology 41 (1991) 479.

[4] T.J. Montine, C.H. Phelps, T.G. Beach, E.H. Bigio, N.J. Cairns, D.W. Dickson, et al., National Institute on Aging-Alzheimer's Association guidelines for the neuropathologic assessment of Alzheimer's disease: a practical approach. Acta Neuropathol 123 (2012) 1-11.

[5] T. Yamamoto, and A. Hirano, A COMPARATIVE STUDY OF MODIFIED BIELSCHOWSKY, BODIAN AND THIOFLAVIN S STAINS ON ALZHEIMER'S NEUROFIBRILLARY TANGLES. Neuropathology and Applied Neurobiology 12 (1986) 3-9.

[6] H. Braak, I. Alafuzoff, T. Arzberger, H. Kretzschmar, and K. Del Tredici, Staging of Alzheimer disease-associated neurofibrillary pathology using paraffin sections and immunocytochemistry. Acta Neuropathol 112 (2006) 389-404.

[7] B.T. Hyman, and J.Q. Trojanowski, Editorial on Consensus Recommendations for the Postmortem Diagnosis of Alzheimer Disease from the National Institute of Aging and the Reagan Institute Working Group o Diagnostic Criteria for the Neuropathological Assessment of Alzheimer Disease. J Neuropathol Exp Neurol 56 (1997) 1095 - 1097.

[8] I.G. McKeith, B.F. Boeve, D.W. Dickson, G. Halliday, J.-P. Taylor, D. Weintraub, et al., Diagnosis and management of dementia with Lewy bodies. Neurology 89 (2017) 88.

[9] A.V. Vatsavayi, J. Kofler, M.A. Demichele-Sweet, P.S. Murray, O.L. Lopez, and R.A. Sweet, TAR DNA-binding protein 43 pathology in Alzheimer's disease with psychosis. Int Psychogeriatr 26 (2014) 987-94.

[10] J.M. Krivinko, S.L. Erickson, Y. Ding, Z. Sun, P. Penzes, M.L. MacDonald, et al., Synaptic Proteome Compensation and Resilience to Psychosis in Alzheimer's Disease. Am J Psychiatry 175 (2018) 999-1009.

# Supplementary Tables

**Supplementary Table 1.** **RNA QC for all subjects**

| **Subject** | **RIN** | **Reads** | **Included** | **Subject** | **RIN** | **Reads** | **Included** | **Subject** | **RIN** | **Reads** | **Included** |
| --- | --- | --- | --- | --- | --- | --- | --- | --- | --- | --- | --- |
| 58 | 6.3 | 3055187 | yes | 2309 | 1.3 | 675341 | yes | 2535 | 2.4 | 1359 | No |
| 297 | 5 | 1056482 | yes | 2320 | 6.2 | 636889 | yes | 2627 | 3.2 | 1020 | No |
| 839 | N/A | 726548 | yes | 2322 | 3.1 | 1036434 | yes | 3647 | 2.2 | 91799 | No |
| 845 | 4.2 | 4074795 | yes | 2324 | 6.9 | 764843 | yes | 3809 | 1.1 | 3005 | No |
| 857 | 3.2 | 1795345 | yes | 2333 | 5.3 | 678496 | yes | 4253 | 2.6 | 1107 | No |
| 879 | 3.4 | 1365370 | yes | 2338 | 6.2 | 743350 | yes |  |  |  |  |
| 1005 | 3.6 | 1117447 | yes | 2369 | 5.7 | 998632 | yes |  |  |  |  |
| 1023 | 2.3 | 1613837 | yes | 2526 | 2.1 | 767648 | yes |  |  |  |  |
| 1049 | 2 | 906495 | yes | 2529 | 3 | 361015 | yes |  |  |  |  |
| 1072 | 4.7 | 1017328 | yes | 2533 | 1.1 | 1309762 | yes |  |  |  |  |
| 1132 | 4.6 | 595274 | yes | 2590 | 1.8 | 846428 | yes |  |  |  |  |
| 1212 | 1.8 | 478645 | yes | 2608 | 5.7 | 796395 | yes |  |  |  |  |
| 1220 | 1.2 | 1087411 | yes | 2613 | 3.8 | 1025752 | yes |  |  |  |  |
| 1375 | 2.7 | 842932 | yes | 2622 | 6.3 | 912757 | yes |  |  |  |  |
| 1510 | 1.6 | 878043 | yes | 2632 | 2.7 | 990039 | yes |  |  |  |  |
| 1564 | N/A | 886787 | yes | 2633 | 3.7 | 820926 | yes |  |  |  |  |
| 1588 | 5.3 | 779649 | yes | 2689 | 5.7 | 573921 | yes |  |  |  |  |
| 1628 | 2.8 | 557694 | yes | 2710 | 2.8 | 654931 | yes |  |  |  |  |
| 1638 | N/A | 684060 | yes | 2775 | 6.2 | 701997 | yes |  |  |  |  |
| 1647 | 5.6 | 961386 | yes | 2788 | 5.5 | 791191 | yes |  |  |  |  |
| 1750 | 3.3 | 827819 | yes | 2810 | 2.5 | 724506 | yes |  |  |  |  |
| 1755 | 2.4 | 601435 | yes | 2815 | 4 | 506823 | yes |  |  |  |  |
| 1789 | 5.5 | 1320672 | yes | 2816 | 5.8 | 1122650 | yes |  |  |  |  |
| 1800 | 3.1 | 886221 | yes | 2904 | 5.5 | 1155713 | yes |  |  |  |  |
| 1828 | 2.3 | 576049 | yes | 2905 | 5.2 | 1078534 | yes |  |  |  |  |
| 1880 | 6.7 | 980102 | yes | 2906 | 3.5 | 680686 | yes |  |  |  |  |
| 1889 | 7.2 | 552354 | yes | 2935 | 3.3 | 1094786 | yes |  |  |  |  |
| 1909 | 3.9 | 865991 | yes | 2982 | 2.9 | 874822 | yes |  |  |  |  |
| 1931 | 2.9 | 735535 | yes | 3040 | 2.2 | 1093797 | yes |  |  |  |  |
| 1942 | 6.9 | 1927829 | yes | 3136 | 4.5 | 1216445 | yes |  |  |  |  |
| 1961 | 4.3 | 1396128 | yes | 3303 | N/A | 600216 | yes |  |  |  |  |
| 1993 | 5.9 | 657576 | yes | 3745 | N/A | 958763 | yes |  |  |  |  |
| 2007 | 6.7 | 2177107 | yes | 3754 | 2.3 | 1141576 | yes |  |  |  |  |
| 2015 | 4.1 | 674238 | yes | 3767 | N/A | 894952 | yes |  |  |  |  |
| 2016 | 2 | 799681 | yes | 3849 | 2.2 | 959940 | yes |  |  |  |  |
| 2062 | 4.8 | 749303 | yes | 4051 | 2.9 | 746652 | yes |  |  |  |  |
| 2073 | 4.5 | 7715622 | yes | 4197 | 2.7 | 1200928 | yes |  |  |  |  |
| 2215 | 3.9 | 853518 | yes | 4328 | 4.8 | 911336 | yes |  |  |  |  |
| 2255 | 2.4 | 939090 | yes | 4538 | N/A | 937471 | yes |  |  |  |  |
| 2292 | 2.6 | 699858 | yes | 4682 | 4.6 | 811656 | yes |  |  |  |  |

**Supplementary Table 2. Missing Rate of Genes Retained for Analysis.**

|  |  | AD+P | | | | | | | | | |
| --- | --- | --- | --- | --- | --- | --- | --- | --- | --- | --- | --- |
|  | Missing Rate Percent | 0 | 1 – 10 | 10 – 20 | 20 – 30 | 30 – 40 | 40 – 50 | 50 – 60 | 60 – 70 | 70 – 80 | 80 - 90 |
| AD-P | 0 | 8402 | 0 | 0 | 0 | 0 | 0 | 0 | 0 | 0 | 0 |
|  | 1 – 10 | 0 | 2248 | 380 | 31 | 2 | 0 | 0 | 0 | 0 | 0 |
|  | 10 – 20 | 0 | 413 | 610 | 188 | 23 | 4 | 1 | 0 | 0 | 0 |
|  | 20 – 30 | 0 | 54 | 335 | 321 | 115 | 37 | 2 | 0 | 0 | 0 |
|  | 30 – 40 | 0 | 6 | 116 | 340 | 296 | 185 | 30 | 4 | 0 | 0 |
|  | 40 – 50 | 0 | 0 | 11 | 114 | 171 | 237 | 141 | 17 | 3 | 0 |
|  | 50 – 60 | 0 | 0 | 3 | 23 | 84 | 222 | 0 | 0 | 0 | 0 |
|  | 60 – 70 | 0 | 0 | 0 | 3 | 27 | 123 | 0 | 0 | 0 | 0 |
|  | 70 – 80 | 0 | 0 | 0 | 0 | 1 | 21 | 0 | 0 | 0 | 0 |
|  | 80 - 90 | 0 | 0 | 0 | 0 | 0 | 2 | 0 | 0 | 0 | 0 |

## Supplementary Table 3A and 3B. Tabulation of Differential Gene Expression Results.

| A. Differentially Expressed Genes Analyzed after Quantile Normalization | | | | | |
| --- | --- | --- | --- | --- | --- |
| p-value | < 1^-5^ | < 1^-4^ | < 1^-3^ | < 1^-2^ | < 5^-2^ |
| DE Genes | 0 | 1 | 16 | 228 | 1077 |

| B. Distribution of Differentially Expressed Genes by Cell Type and p-value | | | | | |
| --- | --- | --- | --- | --- | --- |
| p-value | < 1^-5^ | < 1^-4^ | < 1^-3^ | < 1^-2^ | < 5^-2^ |
| Astrocyte | 0 | 0 | 27 | 252 | 1117 |
| Endothelial Cell | 1 | 1 | 24 | 262 | 1287 |
| Microglia | 0 | 0 | 13 | 145 | 728 |
| Excitatory Neuron | 0 | 2 | 10 | 127 | 604 |
| Inhibitory Neuron | 1 | 2 | 9 | 143 | 635 |
| Oligodendrocyte | 1 | 1 | 12 | 145 | 706 |

## Supplementary Table 4. MEGENA Module Differential Expression Analysis.

| Module | Genes | Individual p-value | Individual log(OR) | Univariate AUC | Univariate R^2^ | Multivariate log(OR) | Multivariate  p-value |
| --- | --- | --- | --- | --- | --- | --- | --- |
| c1_450 | 19 | 0.01 | -14.55 | 0.839 (0.749-0.930) | 0.432 | -48.62 | 0.04 |
| c1_493 | 16 | 0.01 | -9.03 | 0.824 (0.728-0.920) | 0.399 | -29.14 | 0.14 |
| c1_463 | 29 | 0.01 | 9.16 | 0.809 (0.711-0.908) | 0.385 | 76.33 | 0.02 |
| c1_252 | 36 | 0.02 | 7.67 | 0.808 (0.706-0.910) | 0.377 | 62.78 | 0.04 |
| c1_435 | 27 | 0.02 | 11.08 | 0.821 (0.726-0.916) | 0.376 | 49.13 | 0.07 |
| c1_240 | 32 | 0.02 | 8.29 | 0.808 (0.709-0.907) | 0.376 | -0.15 | 0.99 |
| c1_451 | 42 | 0.02 | -9.16 | 0.814 (0.715-0.913) | 0.373 | 30.49 | 0.15 |
| c1_486 | 77 | 0.02 | 8.69 | 0.810 (0.709-0.910) | 0.37 | 24.61 | 0.44 |
| c1_85 | 61 | 0.03 | 9.35 | 0.813 (0.711-0.915) | 0.367 | 1.41 | 0.95 |
| c1_241 | 17 | 0.03 | 7.66 | 0.799 (0.697-0.902) | 0.368 | -13.54 | 0.46 |
| c1_453 | 20 | 0.03 | -8.67 | 0.808 (0.707-0.908) | 0.369 | -58.55 | 0.04 |
| c1_280 | 21 | 0.03 | 7.88 | 0.816 (0.714-0.917) | 0.364 | 33.02 | 0.10 |
| c1_517 | 16 | 0.03 | 8.33 | 0.812 (0.717-0.908) | 0.363 | 90.20 | 0.04 |
| c1_268 | 15 | 0.04 | 7.16 | 0.810 (0.710-0.910) | 0.363 | -9.02 | 0.62 |
| c1_382 | 18 | 0.04 | 7.24 | 0.800 (0.699-0.901) | 0.361 | 45.96 | 0.08 |
| c1_379 | 16 | 0.04 | -7.41 | 0.802 (0.700-0.904) | 0.359 | -15.19 | 0.37 |
| c1_198 | 50 | 0.04 | -8.95 | 0.799 (0.695-0.904) | 0.36 | 120.54 | 0.02 |
| c1_478 | 64 | 0.04 | 7.83 | 0.807 (0.706-0.909) | 0.358 | -34.15 | 0.13 |
| c1_387 | 17 | 0.04 | -7.12 | 0.806 (0.705-0.908) | 0.357 | 12.90 | 0.32 |
| c1_218 | 114 | 0.04 | 7.47 | 0.803 (0.701-0.905) | 0.359 | -44.43 | 0.09 |
| c1_118 | 76 | 0.04 | 7.07 | 0.809 (0.708-0.909) | 0.357 | -116.83 | 0.03 |
| c1_182 | 59 | 0.04 | -8.50 | 0.805 (0.704-0.906) | 0.356 | 116.19 | 0.04 |
| c1_123 | 22 | 0.04 | 7.48 | 0.818 (0.715-0.922) | 0.355 | 32.77 | 0.10 |

# Supplementary Figure Captions

## Supplementary Figure 1. The Effects of Covariates on Gene Expression Are Not Significant.

We tested the covariates (sex, left column; APOE E4, middle column; antipsychotic use, right column) in the following model: RNA expression ~ confounder + psychosis + confounder-by-psychosis interaction. Top row shows the p values for the main effect of each confounder. Bottom row shows the p value for their interaction effects.

## Supplementary Figure 2. Relationship of Excitatory Neuron Proportion to RNA quality meausures.

The relationship of excitatory neuron proportion to RNA-seq read counts and to RIN are shown. There was no significant correlation with read counts. RIN was significantly correlated with excitatory neuron proportion, but this correlation did not differ between AD+P and AD-P subjects and thus would not account for the difference between groups in excitatory neuron proportion (psychosis-by-RIN interaction p=0.41).

## Supplementary Figure 3. Relationship of Excitatory Neuron Proportion to Neuropathologies.

There was a modest association of excitatory neuron proportion with tau, but not Amyloid Beta burden. However, the relationship with tau was not significant in AD+P as a number of subjects have low excitatory neuron proportions despite modest local tau burden (as indicated by our analysis of excitatory neuron proportion which controlled for tau burden). This suggests the presence of additional neurodegenerative mechanisms in AD+P. Other neuropath measures appear protective (IBA labeling- indicative of microglial presence), modestly impairing (HLA:IBA ratio, an indicator of the burden of activated microglia), or unrelated to excitatory neuron proportion (LB, TDP presence, Microvascular Sum Score).

## Supplementary Figure 4. Relationship of Excitatory Neuron Proportion to Other Cell Type Proportions.

The relationship of excitatory neuron proportion to proportions of other cell types are shown. Given that proportions must sum to 1.0, the observed relationships are, as expected, largely inverse.

## 4.4 Supplementary Figure 5. Distribution of DE Genes in AD-P relative to AD+P Subjects.

DE gene results underwent quantile normalization utilizing a regression model which incorporated neuropathological covariates and proportions of endothelial cells, excitatory neurons, and oligodendrocytes. The total number of nominally significant (p < 0.05) DE genes remaining after quantile analysis was limited to 1077, none of which had q < 0.1.

# Supplementary File Captions

## Supplementary File 1. Differentially expressed transcripts without cell type proportion adjustment.

Transcripts are organized with respective Log2 Fold Change (Log2FC), nominal significance (p-value), the difference of RNA transcript levels (Log2) between AD+P and AD-P, and false discovery rate (q-value). Log2FC is a function of AD-P relative to AD+P. Quantile normalization was applied across samples using normalizeQuantiles function from R package Limma.

## Supplementary File 2. Differentially expressed transcripts with cell type proportion adjustment.

Transcripts are organized with respective Log2 Fold Change (Log2FC), nominal significance (p-value), the difference of RNA transcript levels (Log2) between AD+P and AD-P, and false discovery rate (q-value). Log2FC is a function of AD-P relative to AD+P. Quantile normalization was applied across samples using normalizeQuantiles function from R package Limma.

## Supplementary File 3. Differentially expressed transcripts between cell types.

Application of est_frac from R package MIND was used to estimate cell type proportion, in which transcripts found within each cell type was evaluated for differential expression.

## Supplementary File 4. Supplemental File 4. MEGENA Gene Modules.

Gene co-expression network analysis was performed using R package MEGENA. The minimum module size was set to 15 genes. Co-expressed genes were clustered into 288 modules.

## Supplementary File 5. MEGENA Module Strength of Association with Psychosis.

Module eigengenes (MEs) were first tested independently for strength of psychosis prediction (File S5A). A multiple regression model then evaluated the association between MEs and neuropathologic covariates (File S5B). Finally, these analyses were repeated by adding cell type proportions into the logistic regression as covariates (File S5C).

## Supplementary File 6. Correlation between synaptic proteins and transcripts.

Synaptic genes that overlapped from this study and Kivinko et al. (2018). The RNAseq data set was first adjusted using the same ANCOVA as in Krivinko et al. The protein-transcriptome correlation was then evaluated after cell type proportions were adjusted in the RNAseq ANCOVA analysis.
