# Supplementary figures and images for: Psychosis in Alzheimer's Disease Is Associated With Increased Excitatory Neuron Vulnerability and Post-transcriptional Mechanisms Altering Synaptic Protein Levels
Source: Front Neurol. 2022 Mar 2;13:778419. doi: 10.3389/fneur.2022.778419 (PMC8925864; doi:10.3389/fneur.2022.778419)

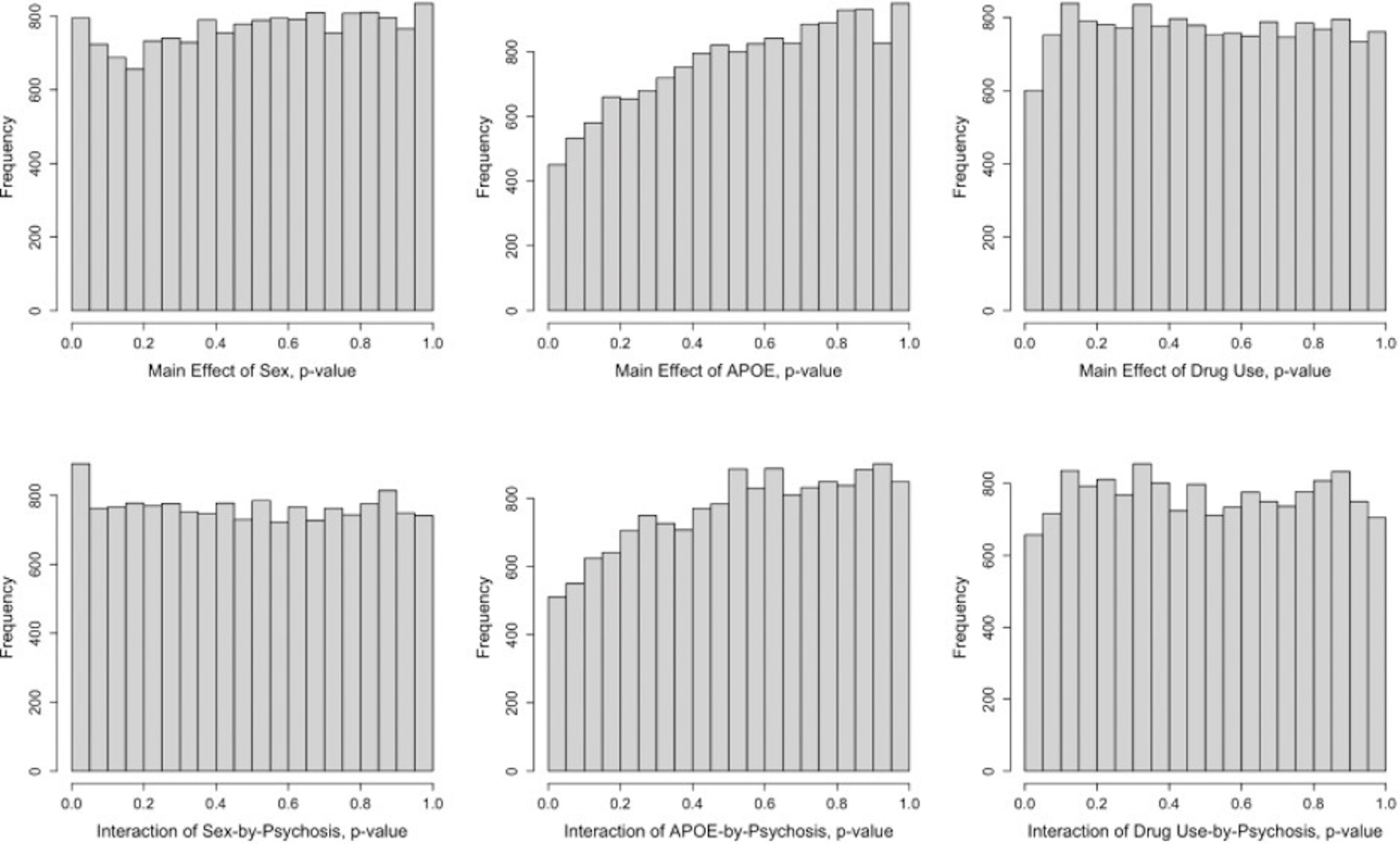

Supplement: Supplementary file 8 [file Image_1.JPEG]

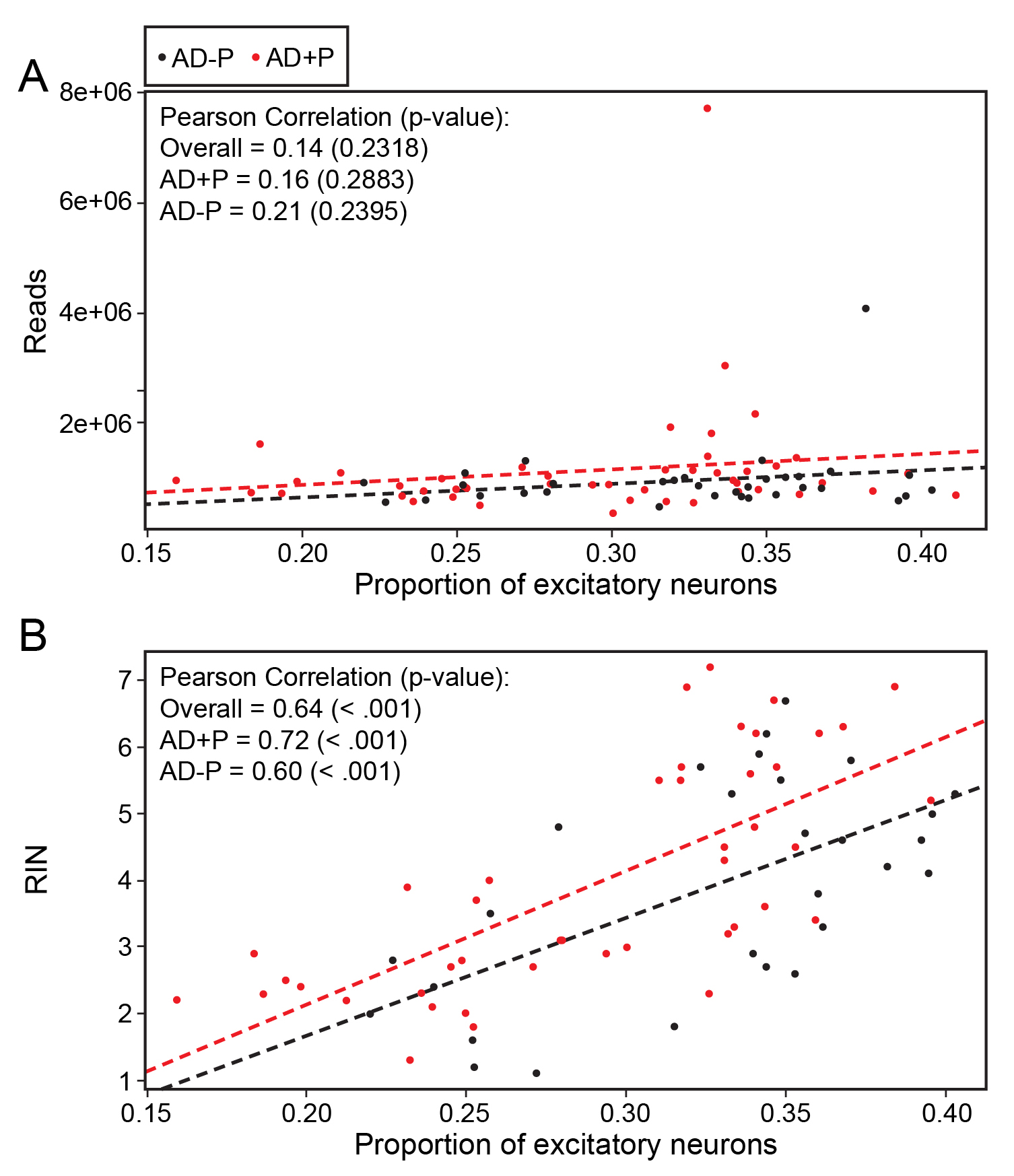

Supplement: Supplementary file 9 [file Image_2.JPEG]

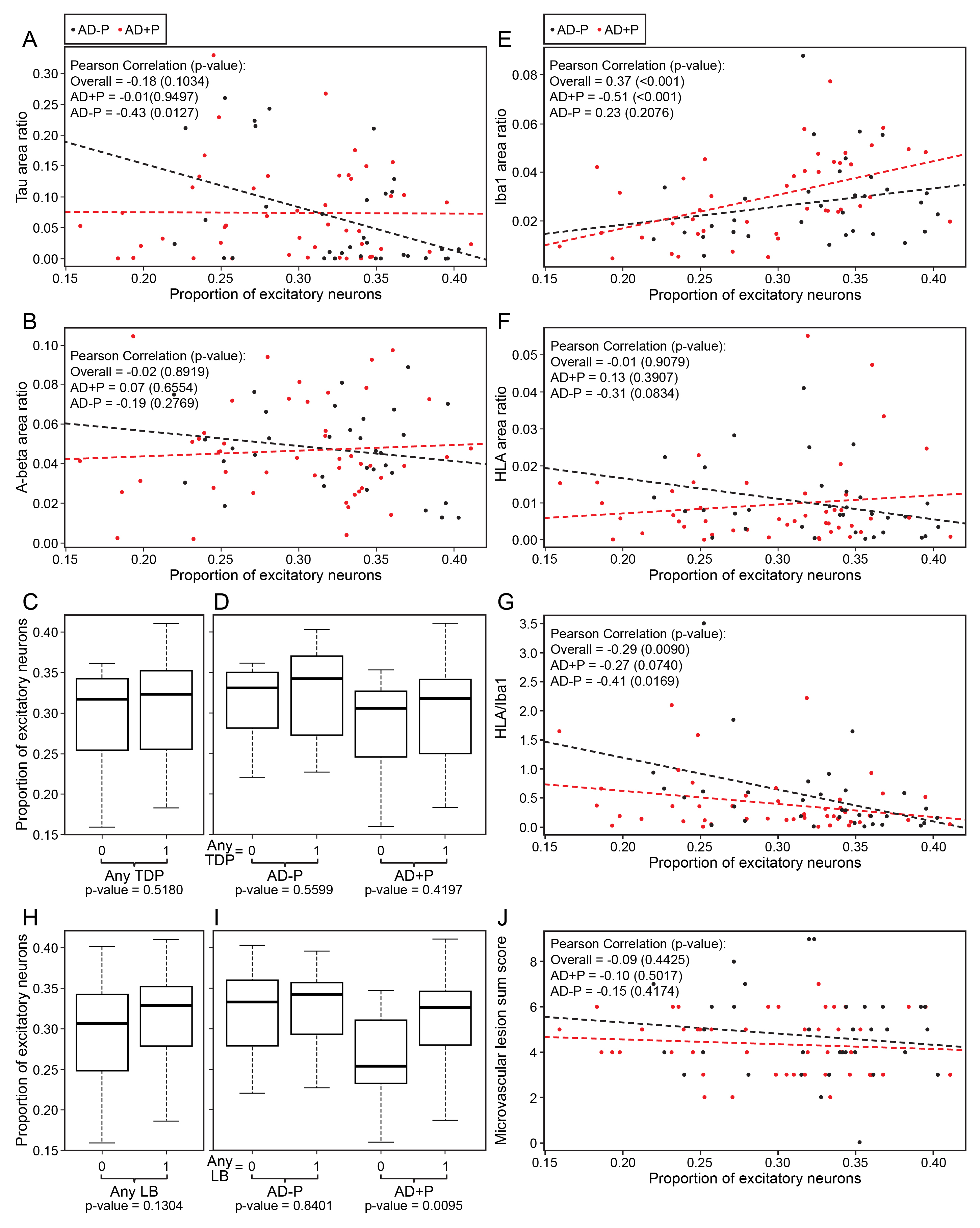

Supplement: Supplementary file 10 [file Image_3.JPEG]

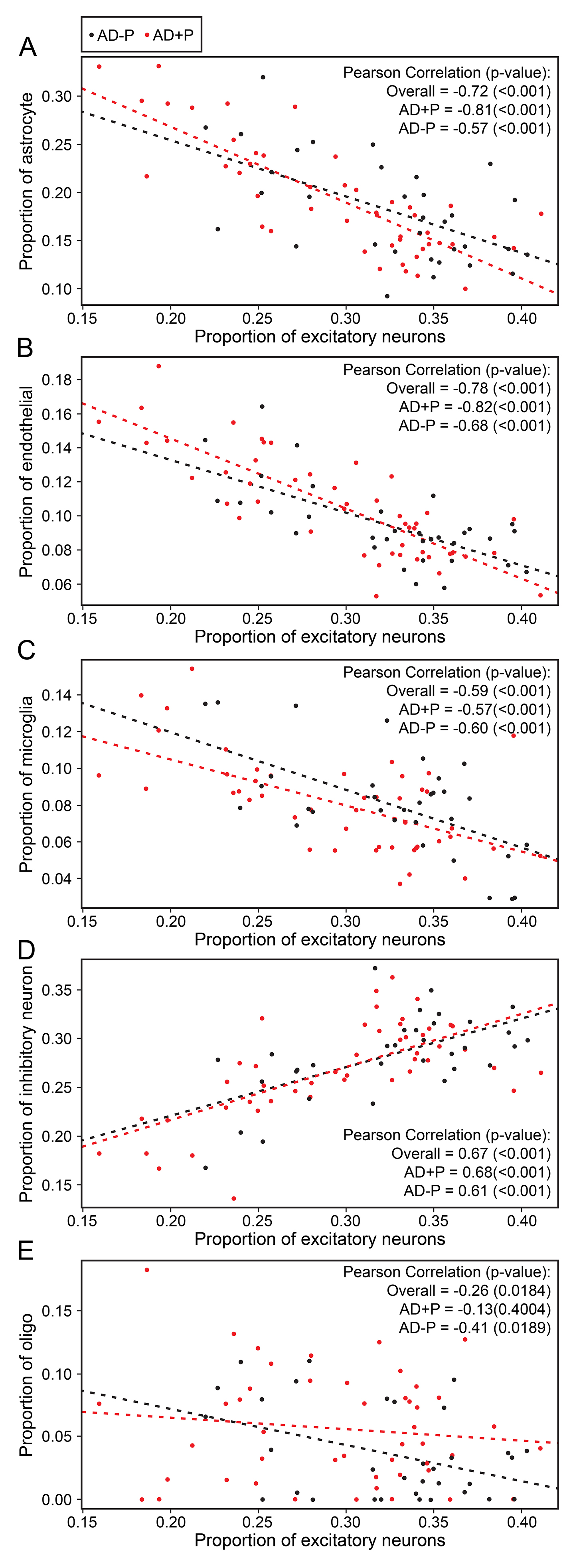

Supplement: Supplementary file 11 [file Image_4.JPEG]

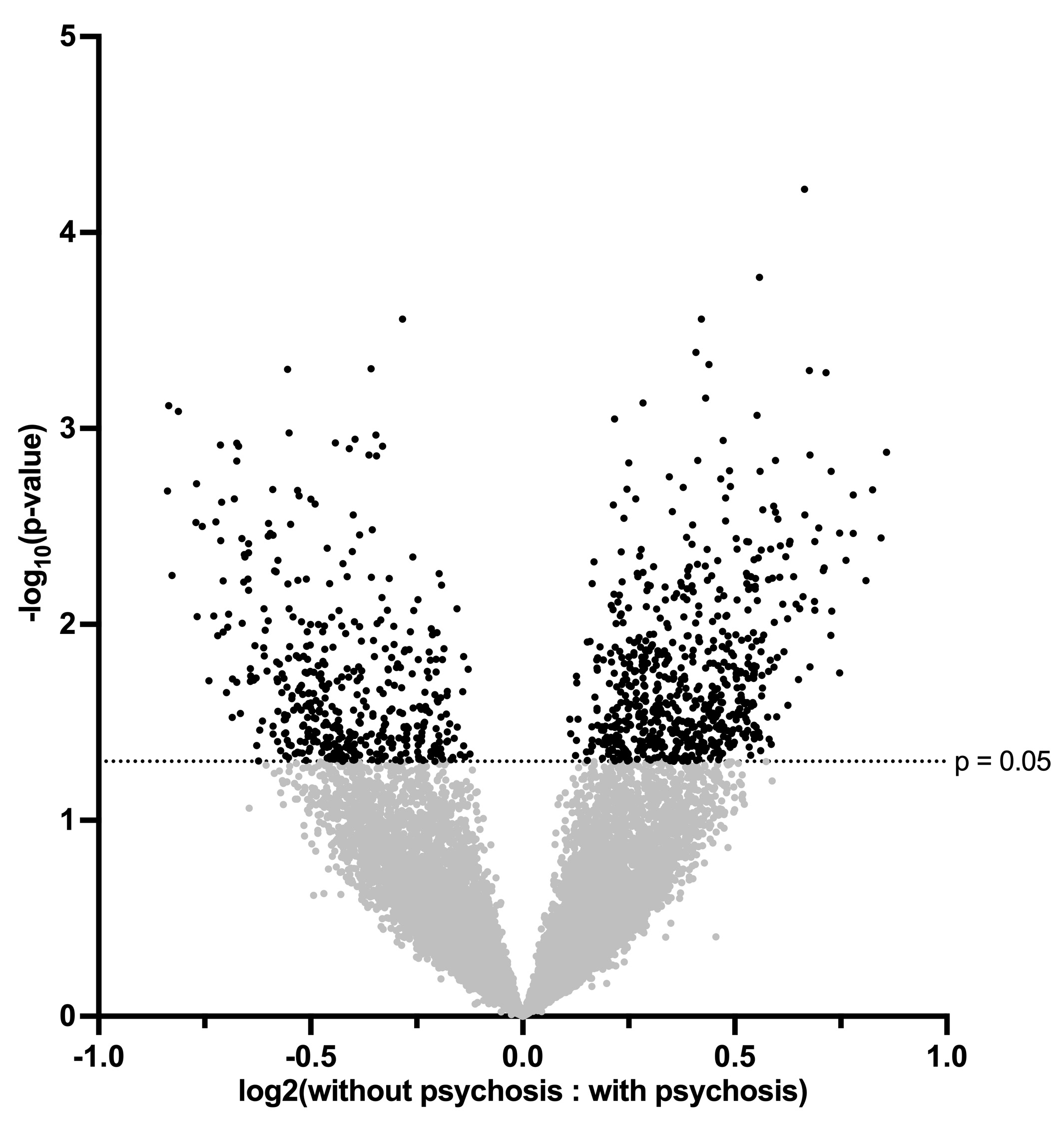

Supplement: Supplementary file 12 [file Image_5.JPEG]
